# A Potential In Vitro 3D Cell Model to Study Vascular Diseases by Simulating the Vascular Wall Microenvironment and Its Application

**DOI:** 10.3390/life12030427

**Published:** 2022-03-15

**Authors:** Yingqian Xu, Jia Deng, Shilei Hao, Bochu Wang

**Affiliations:** 1 Key Laboratory of Biorheological Science and Technology, Ministry of Education, College of Bioengineering, Chongqing University, Chongqing 400030, China; 10818@cqmpc.edu.cn (Y.X.); hao_shilei@126.com (S.H.); 2 Chongqing Engineering Research Center of Pharmaceutical Sciences, Chongqing Medical and Pharmaceutical College, Chongqing 401331, China; 3 Chongqing Key Laboratory of Natural Medicine Research, College of Environment and Resources, Chongqing Technology and Business University, Chongqing 400067, China; jiadeng2011@hotmail.com

**Keywords:** vascular disease, drug screening, shear stress, in vitro model, cell co-culture

## Abstract

Background: Current in vitro vascular models are too simple compared with the real vascular environment. In this research, a novel in vitro 3D vascular disease model that simulated the vascular microenvironment was introduced. Methods: This model was mainly established by low shear stress and co-culture of endothelial cells and smooth muscle cells. Characterization and reproduction of the pathological state of the 3D model were determined. The effect of two clinical drugs was verified in this model. The difference of drug screening between a traditional oxidative-damaged cell model and this 3D model was determined by HPLC. Results: This model presented many disease markers of vascular diseases: abnormal cellular shape, higher endothelial cell apoptotic rate and smooth muscle cell migration rate, decreased superoxide dismutase level, and increased malondialdehyde and platelet-derived growth factor level. The drugs effectively reduced the disease indices and relieved the damage caused by low shear stress. Compared to the traditional oxidative-damaged cell model, this 3D model screened different active components of *Salviae Miltiorrhizae* extract, and it is closer to clinical studies. Conclusions: These results suggest that the 3D vascular disease model is a more efficient and selective in vitro study and drug screening platform for vascular diseases than previously reported in vitro vascular disease models.

## 1. Introduction

Vascular diseases, mainly including atherosclerosis, inflammatory vascular disease, and functional vascular diseases, are the main causes of death in many countries, and the incidence rate and mortality of vascular diseases are increasing rapidly [1]. Therapeutic interventions and drug development of vascular diseases have gained major advancements; however, the pathophysiology study of many vascular diseases is still largely unclear, and new drug discovery and screening is limited due to the complexity of the vascular environment and difficulty of building vascular models. In vitro cell models can narrow the gap between in vitro and in vivo study of vascular diseases [2]. These models have a major role in studying the influence of various factors on vascular diseases [3]. In addition, they are useful for studying drug–drug interaction, drug absorption, distribution, metabolism, excretion, and toxicity testing, or drug screening [4]. Endothelial cells (ECs) are important cells that constitute the internal structure of blood vessels and are crucial for vascular function, so the majority of in vitro vascular models are composed of EC monolayers [5]. However, vascular vessels are multicellular organs [6]. Pathological study or drug screening of vascular diseases based on EC monolayers are sometimes different from in vivo or clinical trials, mainly due to the differences between the in vitro and in vivo environment [7].

The discovery of new and efficacious lead compounds is critical to the continued development of drugs to treat vascular diseases. However, research and development of drugs are time-consuming, cumbersome, and expensive, and identified promising lead compounds often fail at later stages of clinical testing [8]. As a result, the rate of introduction of new and approved drugs into the marketplace has decreased in recent years [9]. These failures represent significant inefficiencies in the drug development process and also result in excessive manpower and material cost. New strategies are clearly needed to screen potential drugs in a manner that mitigates the issues of long development time, high cost, and low success rate. Most new drug candidates of vascular diseases fail in clinical trials because promising lead candidates that possess excellent activates in vitro exhibit low activity in animal or human trials [10]. The main reason for this discrepancy is poorly understood and is a major focus in the field of drug research. It is likely that differences in the biomechanical environment or cellular microenvironment between in vitro and in vivo systems account for the poor activity of some compounds in clinical trials [11]. Liao et al. have proposed a new discipline of biomechanopharmacology to investigate the specific challenges posed by pharmacology and biomechanics [12]. Eukaryotic cells are extremely complex and contain various organelles, cell-surface receptors, and ion channels, among numerous other cellular structures. In addition, differences in the cellular microenvironment can result in alterations in the cellular response to external stimuli [13]. Thus, drugs may have varying therapeutic effects on cells or tissues in different environments and pathological conditions. Cellular receptors or enzymes are attractive targets for pathological research or drug development of vascular diseases, resulting in the development of in vitro vascular models that modulate receptor or enzyme activities at the single-cell level [14]. However, these models, such as the single-cell model, are often simplistic and lack the dynamic environment seen in the internal environment. It is therefore plausible that differences in the biomechanical microenvironment between in vitro drug screening models and in vivo animal experiments account for the failure of some compounds in clinical trials [15]. Taking atherosclerosis as an example, atherosclerosis is the dominant vascular disorder, and new drug research and development to combat atherosclerosis this disease is a top priority [16]. In early stage of atherosclerosis, oscillating or low shear stress (LSS) of blood flow activates the vascular endothelium and results in the production of many pro-atherogenic factors [17,18]. Previous studies indicate that LSS can induce apoptosis and oxidative stress response in ECs and promote the production of pro-atherogenic factors [19,20]. These factors induce SMC migration and proliferation, ultimately resulting in inflammation, vascular lumen thickening, and the formation of atherosclerosis. Many compounds that possess excellent anti-atherogenic effect have been identified in vitro, but few of these compounds translate into efficacious in vivo therapies [21].

The blood vessel is a multicellular organ, consisting of ECs on the inner layer, epithelial cell components, smooth muscle cells (SMCs), and fibroblasts on the outer layer. Although the smooth muscle layer is separated from the endothelium by the internal elastic lamina, numerous membrane pores permit direct communication between SMCs and ECs. The other important factor of vascular microenvironment is shear stress. Shear stress modulates endothelial structure and function and also directly or indirectly affects physiological function of SMCs [22]. However, common in vitro vascular models are based on static single cells, which have difficulty in elucidating some mechanisms of vascular diseases due to microenvironmental differences. Currently several three-dimensional (3D) cell models based on co-culture of ECs and SMCs have been developed, including direct culture of ECs and SMCs, and co-culture on opposite sides of a membrane [23]. These static 3D cell models can preserve physiological cell–cell interaction, but they also poorly mimic the in vivo vascular conditions due to the absence of the physiological level of fluid flow. Some researchers tried replicating the physiological flow of blood vessels on the basis of static 3D cell models, making these models closer to the real internal environment [24]. At present, these dynamic 3D cell models force normal shear stress or strain to simulate the normal physiological flow state of blood vessel. They were mainly used to study the mechanism of cell interactions and signaling, but they are inappropriate for drug screening of vascular diseases. The proposed 3D vascular disease model (VDM) in this manuscript aims to mimic a pathological state of blood vessels by abnormal fluid stress and lay the foundation for drug screening of vascular diseases.

Our previous research has proven that biomechanics could influence cellular function and the ability of cells to absorb drugs [25]. This manuscript introduced an in vitro 3DVDM based on simulating the in vivo vascular microenvironment by co-culture of ECs and SMCs and the biomechanical forces seen in pathological state of blood vessels. In this model, some pathological factors including cell morphology and apoptosis, SMC migration, the production of platelet-derived growth factor (PDGF), superoxide dismutase (SOD), and malondialdehyde (MDA) of ECs were studied to ensure that this model could provide a similar representation of the pathological state of vascular diseases. The effects of a clinical drug trapidil and a traditional Chinese medicine with anti-atherogenic activity, termed *Salviae Miltiorrhizae* (*SM*), were tested in this model [26]. In addition, the active components of *SM* were screened in this model, and the differences in drug screening between the 3D model and a traditional oxidative-damaged cell model were compared. This novel in vitro 3D model should provide an effective tool to study vascular disease and screen new compounds to combat vascular diseases.

## 2. Materials and Methods

### 2.1. Cells and Culture Conditions

Both ECs and SMCs (Beijing BioWin Scientific Development Co., Ltd., Beijing, China) were grown in complete medium RPMI 1640 media with 10% calf serum (Thermo Fisher Scientific China, Co., Ltd., Shanghai, China), 100 U/mL penicillin (Life Technologies, Grand Island, NY, USA), and 100 μg/mL streptomycin (Life Technologies, Grand Island, NY, USA) and were maintained in a humidified cell culture chamber (37 °C, 5% CO_2_). Trapidil was commercially obtained (Shanghai Jimian Co., Ltd., Shanghai, China), and extracts of SM were produced according to previously published methods [27].

### 2.2. Fabrication of the In Vitro 3D VDM

**Figure 1:**
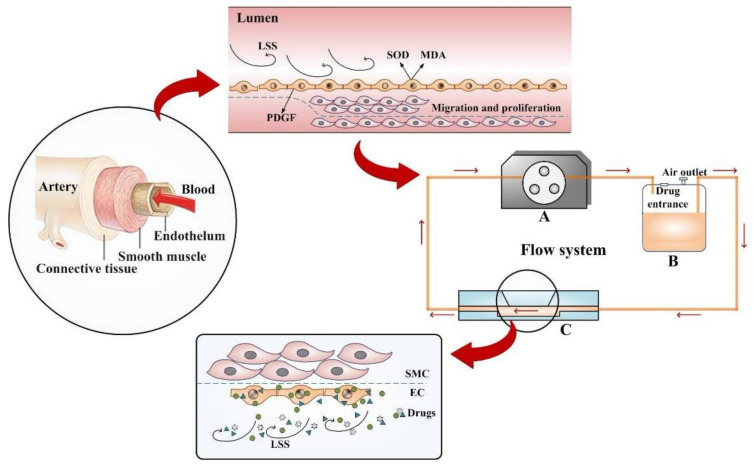
Experimental setup of the drug screening model. The model was inspired by the nosogenesis of early vascular disease caused by LSS. The flow system is available for providing LSS, containing a self-designed parallel flow chamber simulating simplified microenvironment (**C**), a constant flow pump (**A**), and a liquid reservoir (**B**), connected by some silicone tubes.

In order to study the pathology of vascular diseases and screen potentially therapeutic compounds in a system that more closely resembled the microenvironment of the vascular system, an in vitro 3D VDM was generated (Figure 1). This model mainly consisted of three parts: a dynamical system (constant flow pump), a liquid reservoir, and a cell culture system (parallel flow chamber). The constant flow pump recirculated media between the flow chamber and reservoir, as well as provided the motive power to generate shear stress. The reservoir consisted of an inlet and outlet port for media, a port for introduction of drugs, and a filtered air outlet. The air outlet allowed the exchange of 5% CO_2_ between the reservoir and the incubator, thus maintaining appropriate pH in the media. The parallel flow chamber contained a porous polyester membrane on its upper plate, and SMCs and ECs were plated on either side of this membrane. ECs directly communicate with SMCs through the numerous membrane pores of the internal elastic lamina, which separate SMCs from endothelium, and our model simulates this process. The 0.4 μm pores in the membrane allowed for a material and signal interaction between ECs and SMCs [28].

ECs and SMCs were seeded into separate flasks and grown for 48 h to establish confluent monolayers. These cells were then detached from flasks using trypsin (Thermo Fisher Scientific, China, Co., Ltd., Shanghai, China). ECs were seeded by dropwise addition of 1.4 × 10^5^ cells in 0.7 mL of complete media onto the polyester filter (0.4 μm) of a hanging cell culture insert transwell plate (Millipore, Billerica, MA, USA) and adhered for 5 h (37 °C), and then the transwell filters with ECs were transferred to a 6-well plate containing 2 mL complete medium. A total of 2 × 10^5^ SMC cells in 1 mL complete media were then added to the inner transwell compartment, and cultures were incubated for 24 h prior to experimental use. Shear stress were carried out in a modified parallel rectangular flow chamber that was manufactured in our laboratory. Transwell filters containing ECs and SMCs on opposing sides of the filter were then inserted into this flow chamber. A flow of media lacking CS was applied to this chamber with motive power produced by peristaltic pump. It can provide LSS for ECs but not SMCs, which likely simulates physiological conditions found in blood vessel walls. ECs were then exposed to flow rates of 0, 1, 2, 4, 6, 8, or 10 dyn/cm^2^ for 6, 12, or 24 h, respectively, and the theoretical value of shear stress was determined referring to our previous method [25]

**Figure 2:**
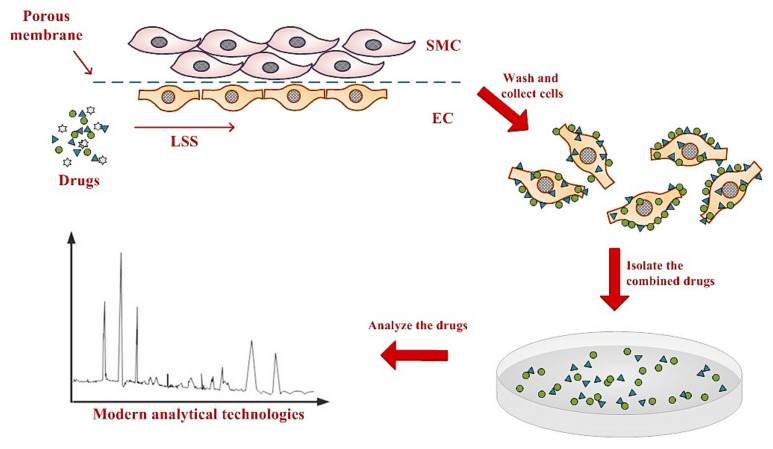
Establishing an anti-vascular disease drug screening model in the mimetic vessel under LSS. SMCs and ECs are seeded on the separate sides of a semi-permeable membrane and transferred to a flow chamber. Cell culture media containing candidate compounds are pumped into the flow chamber and directly interact with ECs but not SMCs. Sufficient time is allowed for binding of candidate compounds to cells. Cells are then harvested, and modern analytical technologies are used to identify drugs that were taken up into ECs and SMCs.

This model also allows for testing the effect of drugs directly on ECs by addition of drugs to the recirculating media. Unbound drugs are then washed out by flowing media without drugs, and absorbed drugs were analyzed by modern analysis and testing technologies (Figure 2).

### 2.3. Cell Viability Assays

ECs and SMCs were grown on transwell filters until cultures reached 90% confluency. Complete media was then removed and replaced with complete media lacking CS. To find proper magnitude and duration of LSS to build a cell model with a pathological state, the samples were then transferred to the flow chamber and incubated at 37 °C for 6, 12, or 24 h under shear stresses of 1, 2, 4, 6, 8, or 10 dyn/cm^2^, respectively. As a control, ECs and SMCs were grown in the absence of LSS (static control cells) for 6, 12, or 24 h, respectively. EC or SMC cell suspensions were collected from either side of the transwell filter and centrifuged (1000 rpm, 5 min, 25 °C), and the cells were uniformly resuspended in the complete medium. A cell counting kit-8 (CCK8) (Beyotime, Shanghai, China) was used to detect the cell viability. Briefly, the CCK8 reagent was added in the cell suspensions in 96-well plates (20 μL/well), and the cells were maintained in the humidified cell culture chamber for 1 h. Then, the optical density (OD) of the samples was determined at 450 nm by a universal microplate spectrophotometer (Bio-Rad). Cell viability can be calculated according to the following formula: Cell Viability (%) = OD_EG_/OD_CG_ × 100%, where OD_EG_ represents the OD value of experiment group and OD_CG_ represents the OD value of control group.

### 2.4. Morphology Analysis of ECs

To study drugs in this model, cells were treated with 250 mM trapidil or 200 μg/mL *SM* extracts. Either trapidil or *SM* was added to complete medium lacking CS, and this medium was added to flow chambers at 37 °C for 12 h under 4 dyn/cm^2^ of LSS. The morphology of ECs was observed by IX71 inverted microscope and phase contrast microscopy (Olympus, Shinjuku, Tokyo, Japan). Micrographs were obtained using a charged-coupled device digital camera 320 (Tykor, Guangzhou, China) and software tImaPro (Tykor, Guangzhou, China). Morphological effects were determined using the method of our previous study [25]. A cell shape index (CSI = 4π × cell area/cells perimeter^2^) reflects the degree of elongation of cells. The value of 1 represents a full circle, and 0 represents a straight line.

### 2.5. Cell Apoptosis Assay

After being exposed to 4 dyn/cm^2^ LSS, ECs were harvested by trypsinization, dilution in phosphate buffer solution (PBS), and centrifugation (1000 rpm, 5 min, 25 °C). Static control samples and drug-treated samples were collected in a similar fashion. Cell apoptosis was tested by Annexin V/PI method analyzed by a FACSvantageSE flow cytometry (BD Biosciences, Shanghai, China), collecting 105 cells per sample.

### 2.6. SMC Migration Assay

A 24-well transwell chamber (Millipore, Billerica, MA, USA) with 8 μm pores was used for the SMC migration assay. SMCs were detached by trypsinization, counted, rinsed with PBS, and centrifuged (1000 rpm, 5 min, 25 °C), and the cells were resuspended in complete media lacking CS (1 × 10^6^ cells/mL). The cells were planted on one side of the transwell filter, and the chemoattractant was the media of the static control cells or cells exposed to LSS. After 12 h of incubation, 95% ethanol was used to fix the SMCs on the filter, and 0.1% crystal violet was used as cell dye. Cells were counted by phase contrast microscopy at a magnification of 200× (Olympus, Shinjuku, Tokyo, Japan), and three randomly chosen fields were evaluated per filter.

### 2.7. Determination of PDGF, SOD, and MDA

Supernatants from static control co-culture cells or co-culture cells exposed to shear stress were collected for quantitative analysis of PDGF, SOD and MDA. A human PDGF enzyme-linked immunosorbent assay kit (Shanghai Saimo Biological Science and Technology Co., Ltd., Shanghai, China) was used to measure PDGF concentration. The OD was measured using a series of PDGF standards of known concentration. A standard curve and a corresponding linear regression equation were created through these values. This equation was then used to convert the OD450 values for each experimental sample into PDGF concentrations. The levels of SOD and MDA were measured by test kits, respectively.

### 2.8. Generation of EC Cellular Extracts after SM Extract Treatment

ECs were treated with *SM* extract (200 μg/mL, 12 h), and then 2 mL of 75% ethanol was used to denature the ECs and extract cellular contents. Samples were then subjected to ultrasonic vibrations by the ultrasonic cell disruption system (Scientz, Ningbo, Zhejiang, China) and centrifuged. The supernatant was condensed by vacuum freeze drier (Thermo Fisher Scientific, China, Co., Ltd., Shanghai, China), dissolved in 70% methanol, and filtered by nylon membrane (0.45 μm, Xingya, Shanghai, China) before high performance liquid chromatography (HPLC) analysis. In the static group, ECs were damaged by 300 μM H_2_O_2_ for 0.5 h and treated with 200 μg/mL *SM* extracts for 12 h. The samples of the static oxidative (H_2_O_2_)-damaged model were prepared using the same methods of the other group.

### 2.9. HPLC Analysis

The samples of *SM* extracts, mixed standards of *SM*, untreated ECs, ECs treated with oxidative stress and *SM* in the static model, and ECs treated with LSS and *SM* in the 3D VDM were collected for HPLC. The conditions for chromatographic analysis have been previously described [29]. Peaks were then identified in the HPLC chromatograph, using a peak area that is at least 3% of the protocatechuic aldehyde as a cutoff for identification.

### 2.10. Statistical Analysis

All results were expressed as mean ± standard deviation (SD), and for all data, one-way analysis of variance assuming equal variance followed by unpaired Student t-tests were used to determine statistical significance calculated by SPSS software (IBM, Armonk, New York, NY, USA).

## 3. Results and Discussion

### 3.1. Increased Shear Stress Correlates with Decreased EC Viability

**Figure 3:**
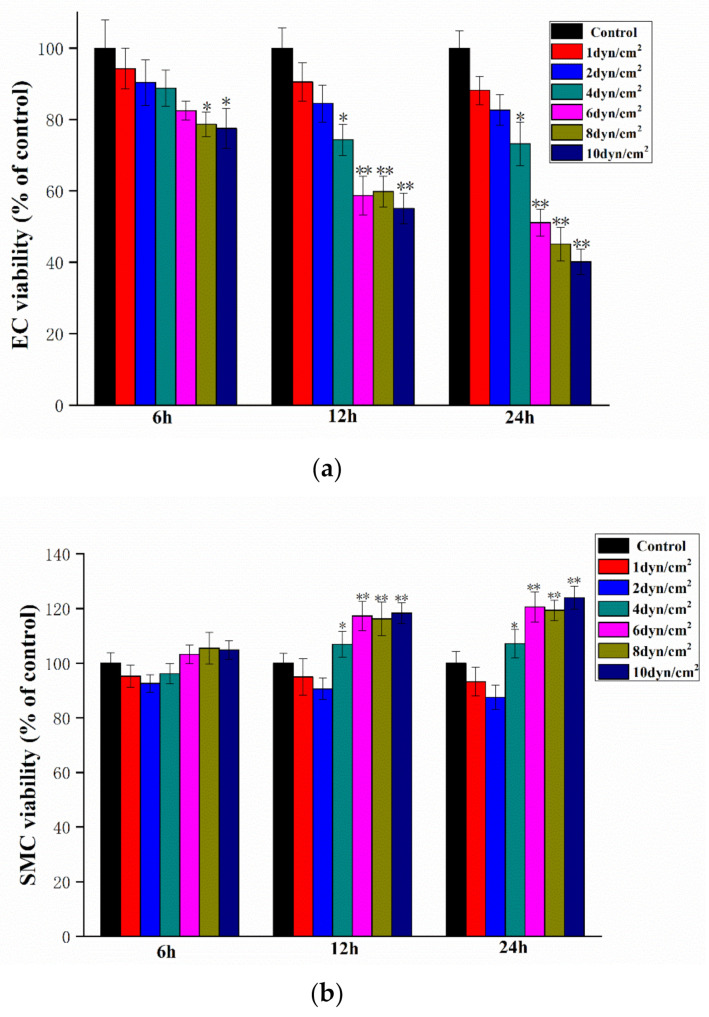
The effect of shear stress rates and time on EC (**a**) and SMC (**b**) viability (compared with the control group, * *p* < 0.05, ** *p* < 0.01, *n* = 5).

In order to determine appropriate levels of shear stress and exposure times, several shear stress rates and exposure times on ECs and SMCs were screened in this model. Either ECs or SMCs were forced by shear stress at the levels of 0, 1, 2, 4, 6, 8, or 10 dyn/cm^2^ and exposure times of 6, 12, or 24 h, respectively, prior to determining cellular viability. Increased shear stress, as well as increased exposure time, resulted in decreased EC viability (Figure 3a). After 6 h of shear stress treatment, the decline in EC viability was not significant compared to the static control samples until the shear stress reached 8 dyn/cm^2^. After 12 h, shear stresses greater than 2 dyn/cm^2^ resulted in significant decreases in cell viability. A total of 24 h of shear stress treatment resulted in similar decreases in EC viability as that of 12 h of shear stress treatment.

The effect of shear stress rates and exposure times on SMC viability was also examined. After 6 h of treatment, no shear stress levels resulted in significant differences in viability as compared to the static control samples. In contrast, shear stress of more than 4 dyn/cm^2^ resulted in significant increases in SMC viability when administered for 12 or 24 h (Figure 3b). Therefore, shear stress conditions of 4 dyn/cm^2^ and 12 h exposure were chosen for the follow-up experiments since these conditions represented the proper shear stress and exposure time needed to obtain significant effect on EC and SMC viability.

### 3.2. Characterization of the 3D VDM by Morphological Analysis of ECs

**Figure 4:**
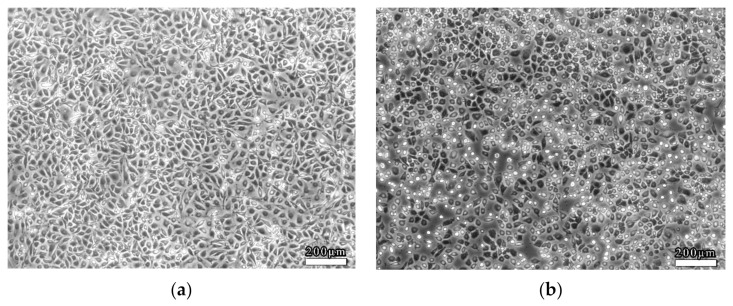
Morphological observations of ECs. ECs were seeded on semi-permeable membranes and exposed to (4 dyn/cm^2^) LSS for 12 h prior to observation by phase contrast microscopy. (**a**) Static control ECs not treated with LSS. (**b**) ECs that were treated with LSS. Scale bars denote 200 μm.

EC viability was determined accompanied by morphological changes after dealing with LSS in the 3D VDM. EC monolayers were treated with shear stress (4 dyn/cm^2^, 12 h) prior to morphological observation by phase contrast microscopy. As a comparison, ECs of the static control group that were not treated with shear stress were closely packed and of fusiform shape (Figure 4a). In contrast, LSS treatment of ECs in the 3D VDM caused shrinkage and rounding of cells in the monolayer, resulting in cells that appeared loosely arranged compared to the static control cells (Figure 4b). In order to quantitatively determine the cellular shape of the 3D VDM, micrographs of the static control cells and the EC monolayers treated with LSS were analyzed by image analysis software. CSI was quantified from these micrographs. The CSI value of static control ECs was approximately 0.6, while the CSI of ECs exposed to LSS of the 3D VDM significantly increased to approximately 0.9.

### 3.3. Reproduction of the Pathological State of Vascular Disease in the 3D VDM

PDGF, SOD, and MDA are chemotactic molecules often released in response to cellular injury. We therefore sought to investigate whether these molecules induced chemotaxis in this model. Tissue culture supernatants from the static control co-culture cells and the cells of the 3D VDM were examined for PDGF levels. The level of PDGF was significantly increased in media from cells exposed to LSS in the 3D VDM as compared with the static condition (*p* < 0.01). Treatment of the cells with LSS in the 3D VDM decreased SOD levels by 50.80% (*p* < 0.01) and increased intracellular MDA levels by 63.27% compared with the static control group (*p* < 0.01) (Table 1).

**Figure 5:**
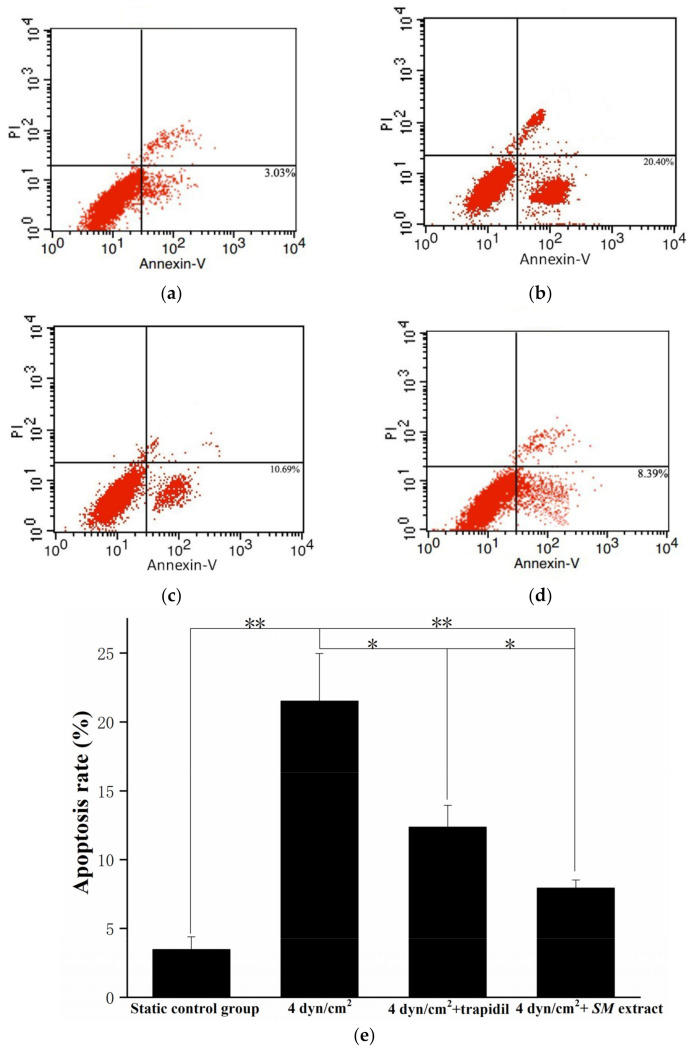
EC apoptosis detection by flow cytometry. (**a**) denotes the apoptosis of the static control group; (**b**) denotes the cell apoptosis of LSS group; (**c**) denotes the cell apoptosis of LSS and trapidil group; (**d**) denotes the cell apoptosis of LSS and *SM* group. Percentages refer to the percent of cells that are Annexin V positive. (**e**) Graphical representation of (**a**–**d**) (* *p* < 0.05, ** *p* < 0.01, *n* = 5).

For further study of the mechanism by which LSS results in decreased EC viability, we examined whether LSS induced apoptosis in ECs (Figure 5). After being exposed to LSS, ECs were collected for cell apoptosis analysis. ECs of the 3D VDM had a higher apoptosis rate as compared to the static control cells, with 21.50 ± 3.47% and 3.47 ± 0.91% of cells positive for Annexin V-FITC, respectively.

**Figure 6:**
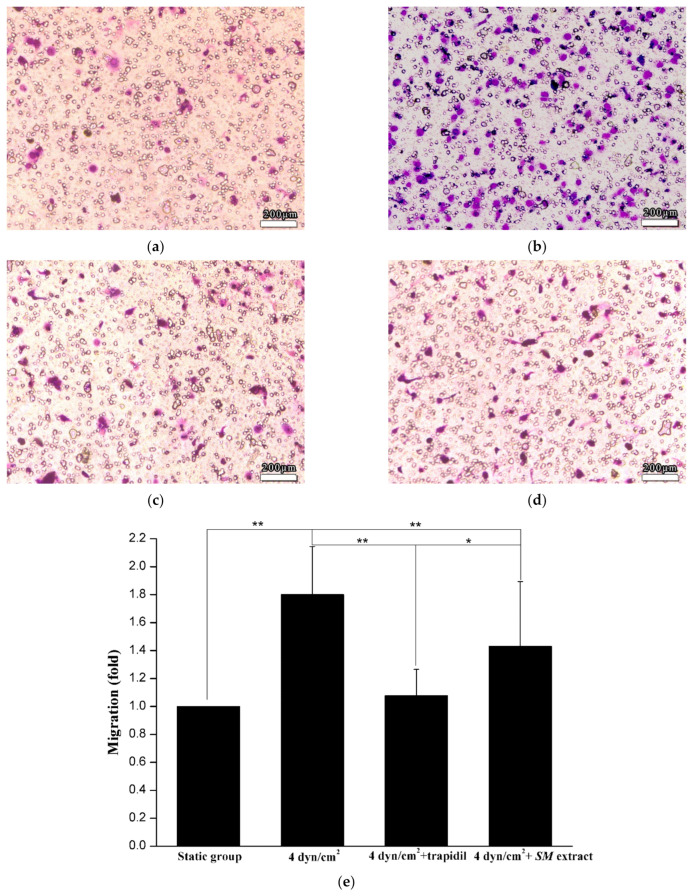
SMC migration assay. (**a**) denotes SMC migration by the culture of static control cells; (**b**) denotes SMC migration by the culture of the cells exposed to LSS; (**c**) denotes SMC migration by the culture of the cells exposed to LSS and trapidil; (**d**) denotes SMC migration by the culture of the cells exposed to LSS and *SM* extracts. Scale bars denote 200 μm. (**e**) Graphical representation of (**a**–**d**). (Compared with the LSS group, * *p* < 0.05, ** *p* < 0.01, *n* = 5).

Tissue culture media from the co-culture cells of the static control group or the 3D VDM were used as chemoattractant for SMC migration, and SMC migration was examined using transwell assays (Figure 6). The results showed that the medium from the cells exposed to the 3D VDM enhanced SMC migration by about 1.8-fold compared with the static control group.

### 3.4. Drug Effect of Trapidil or SM Extract in the 3D VDM

PDGF, SOD, and MDA of tissue culture supernatants from the co-culture cells treated with trapidil or *SM* extract in the 3D VDM were examined (Table 1). The media from cells not treated with drugs or treated with trapidil in the 3D VDM exhibited significantly lower levels of PDGF (*p* < 0.01), with levels similar to the static control group. In contrast, treatment of cells with *SM* extract in this model did not reduce PDGF levels. Additionally, PDGF levels in the *SM* treated group were much higher than that of trapidil treated sample. Treatment of the cells with *SM* extract resulted in lower levels of MDA production by 60.24%, and SOD activity was restored by 43.36% (*p* < 0.01), relative to the cells not treated with drugs in the model. However, cells that were treated with trapidil did not significantly alter the activity of SOD or MDA production in the model.

**Figure 7:**
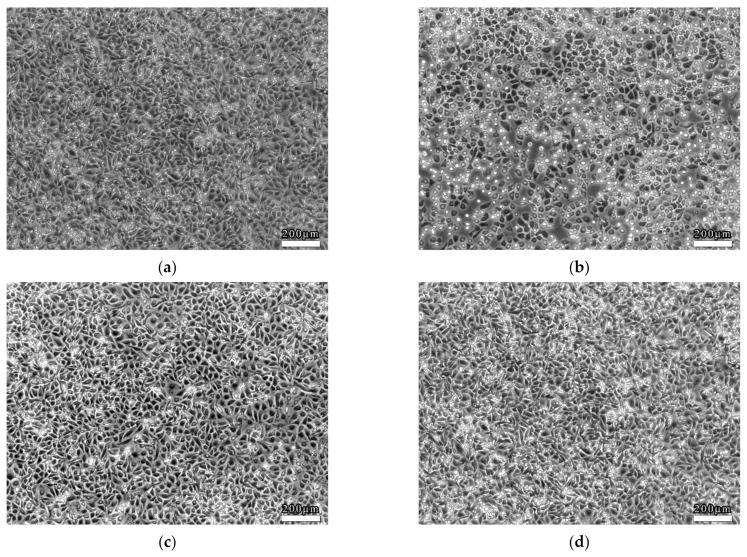

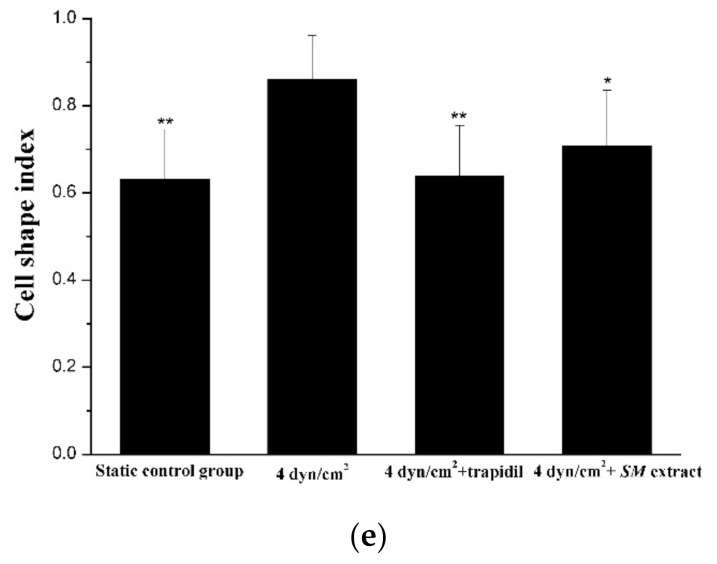
Drug effect on EC morphology. (**a**) denotes the morphology of the static control ECs; (**b**) denotes the morphology of the ECs that were treated with LSS; (**c**) denotes the morphology of the ECs that were treated with trapidil exposed to LSS; (**d**) denotes the morphology of the ECs that were treated with *SM* exposed to LSS. Scale bars denote 200 μm. (**e**) Quantitative analysis of EC shape under static condition or exposure to LSS. Image analysis was used to quantify the cellular shape of ECs after LSS treatment. A static control sample was not subjected to LSS. Samples were also treated with trapidil or *SM* extracts during LSS treatment. Each sample contains analysis on 100 cells (compared with the LSS group, * *p* < 0.05, ** *p* < 0.01, *n* = 5).

The cells treated with trapidil or *SM* extracts in the 3D VDM had a CSI closer to that of the static control cells, indicating more fusiform cells, demonstrating that drug treatment restored cellular shape in the model. These data were consistent with the cellular shapes observed by microscopy, demonstrating that trapidil and SM extracts protect ECs from the harmful effects of LSS in the model (Figure 7).

Treatment of ECs with trapidil reduced LSS-induced apoptosis by 1.7-fold and *SM* extract treatment reduced apoptosis by 2.7-fold, with 12.35 ± 1.60% and 7.93 ± 0.6% of cells positive for Annexin V-FITC, respectively (Figure 5c,d). *SM* extract proved to be more protective against the effects of LSS, with an apoptosis rate that was ~35.79% lower than that of the cells treated with trapidil. In addition, the ability of the medium containing trapidil or *SM* extract to induce SMC migration lower than that of the medium under LSS without drugs by 40% and 20%, respectively (Figure 6c,d).

### 3.5. Bioactive Components Screening of SM Extract in the 3D VDM

**Figure 8:**
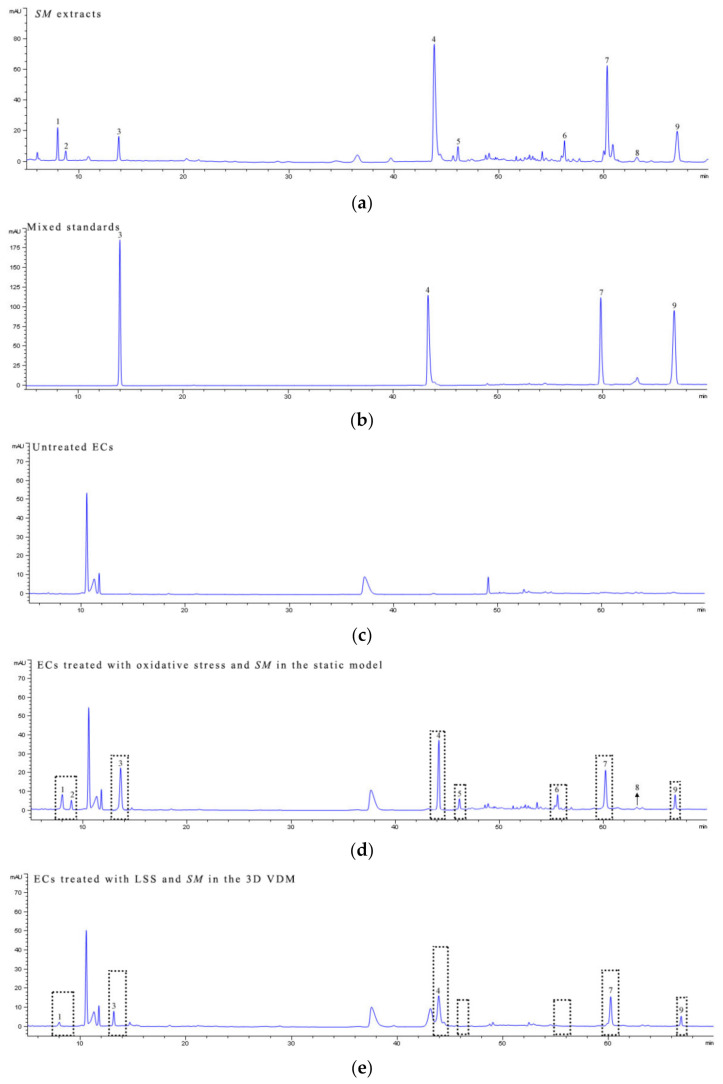
HPLC analysis. ECs were treated with the following conditions prior to harvesting and analysis by HPLC. (**a**) denotes the HPLC chromatogram of the *SM* extracts; (**b**) denotes the HPLC chromatogram of mixed standards; (**c**) denotes the HPLC chromatogram of untreated ECs; (**d**) denotes the HPLC chromatogram of ECs treated with oxidative stress and *SM* in a static model; (**e**) denotes the HPLC chromatogram of ECs treated with LSS and *SM* in the 3D VDM. mAU refers to milli-absorbance units measured at 281 nm.

We next sought to determine the difference of *SM* extract active component screening between the 3D VDM and a traditional static oxidative-damaged cell model. *SM* extract is a complex mixture with numerous constituents, and a total of nine major peaks were obtained by HPLC analysis of *SM* extracts (Figure 8a). Among the nine peaks, four were identified using commercial standards and five were deduced following previously published methods (Figure 8b; Table 2). ECs of the 3D VDM or the static oxidative-damaged cell model were treated with *SM* extract for 12 h. The cells were then collected and analyzed by HPLC. Normal ECs treated with the same procedure were subjected to HPLC analysis as the control (Figure 8c) to the experimental groups. By comparing to the HPLC chromatograph of the normal control ECs, the ECs of the static oxidative-damaged cell model were detected containing nine components of *SM* extract (Figure 8d), and the nine components were, respectively, salvianolic acid B, danshensu, protocatechuic acid, dihydrotanshinone I, protocatechuic aldehyde, salvianolic acid A, cryptotanshinone, methylene tanshiqunone, and tanshinone IIA (peaks 1–9). In contrast, only five components of *SM* extract were identified in the ECs of the 3D VDM, and the five components were, respectively, danshensu, protocatechuic aldehyde, salvianolic acid B, cryptotanshinone, and tanshinone IIA (peaks 1, 3, 4, 7, and 9) (Figure 8e), and these peaks have a smaller area than the corresponding peaks of the chromatograph of the static oxidative-damaged cell model (Figure 8d). The data indicated that ECs of the 3D VDM absorbed both fewer compounds from the *SM* extract and a lower concentration of these compounds, as compared to ECs of the traditional static oxidative-damaged cell model.

## 4. Discussion

In this research, we presented an in vitro 3D cell model to study vascular disease and drug screening. This model was made by simulating the effect of communication between different cell types of the blood vessel wall considering the effect of vascular shear stress. The arterial wall mainly consists of ECs and SMCs, and the interaction between the two cells influences the vascular physiology and pathology. LSS stimulates vascular cells, leads to vascular injury, and may affect drug absorption. The characteristics of the 3D VDM are that ECs are exposed to continuous LSS, ECs can communicate with SMCs through a porous membrane, and drugs are transported to cells by continuous circulation of cell culture media. Taken together, the advantage of this model is that it may simulate many aspects of the microenvironment of vessel walls and may improve the ability of researchers to predict the effects of drugs on target cells.

Shear stresses in arteries range from ~20 to ~60 dyn/cm^2^, and LSS refers to the shear stress that is approximately < 10 to 12 dyn/cm^2^ and unidirectional at any given point (Chatzizisis et al. 2007). Long term exposure to LSS decreases EC viability, while at the same time inducing SMC proliferation. We found that shear stress of 4 dyn/cm^2^ and exposure time of 12 h resulted in significant decrease in EC viability, demonstrating that this model is suitable to recapitulate the microenvironment of the vessel wall of vascular diseases.

Although many in vitro vascular models have previously been built in other researches, most of these models could not accurately reflect a pathological state of vascular diseases. Additionally, it is unclear whether established drug treatments were efficacious to reduce pathological symptoms in these models [30]. We sought to overcome these limitations by inducing a pathological state in the 3D VDM and to determine the effect of drug treatment on cell pathology. LSS induces numerous effects in vivo, including alteration to cellular morphology, cell apoptosis, production of PDGF, SOD, and MDA, and SMC migration. Since these effects can be quantitatively and reproducibly measured, these markers were used to evaluate the reproduction of the pathological state of vascular disease in the 3D VDM. 

In addition, we sought to verify the pharmaceutical effect of drugs in the 3D VDM using the drugs that have been reported to alleviate vascular diseases. Trapidil is a widely used anti-vascular disease drug, and treatment results in highly selective inhibition of PDGF production and SMC migration [31]. SM is a multi-purpose traditional Chinese medicine, which has been used to treat a variety of clinical diseases including vascular diseases [32]. Recent studies indicated that SM exhibited obvious antioxidant activities. These properties make trapidil and SM attractive choices to validate the 3D VDM. The study demonstrated that trapidil protected cellular shape and inhibited EC apoptosis, SMC migration, and PDGF production; however, the drug had no significant effect on the production of SOD and MDA under conditions of LSS in the 3D VDM. The results also showed that the SM extract was able to maintain cellular shape, inhibit cell apoptosis and migration, decrease MDA production, and increase SOD activity; however, it had no effect on the production of PDGF. Thus, trapidil and SM extract treatment verified that the 3D VDM was useful in testing compounds that reduce pathologic effects.

Extracts of SM contain numerous active components including danshensu, protocatechuic aldehyde, protocatechuic acid, salvianolic acid A, salvianolic acid B, dihydrotanshinone I, cryptotanshinone, methylene tanshiqunone, and tanshinone IIA. These compounds exhibit numerous pharmacological effects and act on many cellular targets [33]. The anti-vascular disease activity of SM is well-known and includes improvement of microcirculation and prevention of myocardial ischemia [34]. As a result, treatment with SM extract offers an attractive way to highlight differences of drug screening between the 3D VDM and the traditional static oxidative-damaged vascular disease model. The HPLC results showed that the ECs of the static oxidative-damaged cell model absorbed all the nine components of the SM extract described above (Figure 8a,c), but only five of these compounds were detected in the cells of the 3D VDM (Figure 8e). Our previous study has demonstrated that shear stress could affect drug absorption of cells, and the results in this study support this finding. Protocatechuic aldehyde, danshensu, tanshinone IIA, and salvianolic acid B are able to inhibit many vascular diseases and are clinically effective for the prevention of angina, coronary heart disease, stroke, or other vascular diseases [35]. However, protocatechuic acid, dihydrotanshinone I, and methylene tanshiqunone have no obvious anti-vascular disease effect [36]. The ECs of the static oxidative-damaged cell model absorbed most components of the SM extract including anti-vascular disease components and others having no anti-vascular disease effect, while the ECs of the 3D VDM mainly absorbed the anti-vascular disease active ingredients of SM extract, indicating that the 3D VDM has a higher selectivity for anti-vascular-disease drugs than the traditional static in vitro model.

There are several possible scenarios to explain the differences in SM extract absorption in the 3D VDM and the static oxidative-damaged cell model. LSS and co-culture of ECs and SMCs may result in a different disease state than the oxidative stress model, and the 3D VDM may more accurately reflect the real physiological and disease state seen in vascular diseases. In addition, oxidative stress results in a relatively limited cellular response to damage, and LSS may upregulate numerous cellular receptors and cellular damage response genes. Finally, since SM extracts were administered under flow condition, it likely that differences in affinity and uptake kinetics of SM extract components contribute to the differences seen.

## 5. Conclusions

The researches show that the 3D VDM has potential for the pathophysiology study and efficient drug screening of vascular diseases. The cell co-culture and LSS treatment of the 3D VDM make it closer to the physiological and pathological state of vascular diseases and simulate the microenvironment of blood vessel wall. This model is also able to eliminate some irrelevant compounds in the early state of drug screening of vascular diseases, which will contribute to cost-saving in the drug research process.

## Figures and Tables

**Table 1:** Production of SOD and MDA.

Treatment	SOD, U/mg Protein	MDA, nmol/mg Protein	PDGF, ng/mL
Control	43.62 ± 4.31 **	0.54 ± 0.07 **	3.98 ± 0.57 **
LSS	21.46 ± 2.70	1.47 ± 0.19	14.51 ± 2.90
LSS + trapidil	26.15 ± 2.92	1.28 ± 0.14	5.91 ± 0.42 **
LSS + *SM* extract	37.89 ± 3.65 **	0.72 ± 0.11 **	12.31 ± 1.45

Compared with treatment of LSS, ** *p* < 0.01, *n* = 5.

**Table 2:** Identification of bioactive compounds from *SM* extract.

Peak	Retention Time, Min	Identified or Deduced Compound
1	7.912	Danshensu
2	8.768	Protocatechuic acid
3	13.923	Protocatechuic aldehyde
4	44.043	Salvianolic acid B
5	46.223	Salvianolic acid A
6	56.312	Dihydrotanshinone I
7	60.314	Cryptotanshinone
8	63.313	Methylene tanshiqunone
9	67.117	Tanshinone IIA

## Data Availability

The data presented in this study are available in this article.
